# Cardiopulmonary resuscitation duration and favorable neurological outcome after out-of-hospital cardiac arrest: a nationwide multicenter observational study in Japan (the JAAM-OHCA registry)

**DOI:** 10.1186/s13054-022-03994-2

**Published:** 2022-05-02

**Authors:** Tasuku Matsuyama, Bon Ohta, Kosuke Kiyohara, Tetsuhisa Kitamura

**Affiliations:** 1 Department of Emergency Medicine, Kyoto Prefectural University of Medicine, Kyoto, Japan; 2 Department of Food Science, Otsuma Women’s University, Tokyo, Japan; 3 Division of Environmental Medicine and Population Services, Department of Social and Environmental Medicine, Graduate School of Medicine, Osaka University, Osaka, Japan

**Keywords:** Out-of-hospital cardiac arrest, Cardiopulmonary resuscitation, Cardiopulmonary resuscitation duration, Neurological outcomes

## Abstract

**Objective:**

We aimed to assess the association between cardiopulmonary resuscitation (CPR duration) and outcomes after OHCA.

**Methods:**

This secondary analysis of a prospective, multicenter, observational study included adult non-traumatic OHCA patients aged ≥ 18 years between June 2014 and December 2017. CPR duration was defined as the time from professional CPR initiation to the time of return of spontaneous circulation or termination of resuscitation. The primary outcome was 1-month survival, with favorable neurological outcomes defined by cerebral performance category 1 or 2. We performed multivariable logistic regression analysis to investigate the association between CPR duration and favorable neurological outcomes. We also investigated the association between CPR duration and favorable neurological outcomes stratified by case features, including the first documented cardiac rhythm, witnessed status, and presence of bystander CPR.

**Results:**

A total of 23,803 patients were included in this analysis. Multivariable logistic regression analysis demonstrated that the probability of favorable neurological outcomes decreased with CPR duration (i.e., 20.8% [226/1084] in the ≤ 20 min group versus 0.0% [0/708] in the 91–120 min group, *P* for trend < 0.001). Furthermore, the impact of CPR duration differed depending on the presence of case features; those with shockable, witnessed arrest, and bystander CPR were more likely to achieve favorable neurological outcomes after prolonged CPR duration > 30 min.

**Conclusion:**

The probability of favorable neurological outcome rapidly decreased within a few minutes of CPR duration. But, the impact of CPR duration may be influenced by each patient’s clinical feature.

**Supplementary Information:**

The online version contains supplementary material available at 10.1186/s13054-022-03994-2.

## Introduction

Sudden cardiac arrest in the out-of-hospital setting is a major public health problem in the industrialized world [1–3]. Despite improvements in the quality of resuscitation practices, the proportion of favorable neurological outcomes after out-of-hospital cardiac arrest (OHCA) remains low [1–3]. Early successful return of spontaneous circulation (ROSC) by early interventions, such as “early cardiopulmonary resuscitation (CPR)” and “rapid defibrillation,” is the most important contributor to increasing the rate of favorable neurological outcome after OHCA [1, 2].

During CPR, one of the biggest challenges is determining whether to continue or terminate resuscitation efforts when OHCA patients do not respond to conventional CPR.

A CPR duration of 20–30 min is considered an important cutoff point [2, 4, 5]. However, some studies have suggested that although the probability of favorable outcome decreased with increasing CPR duration, some patients might benefit from longer resuscitation efforts than have been previously reported [6–9]. A study on in-hospital cardiac arrest demonstrated that longer CPR duration might result in increased return of spontaneous circulation (ROSC) and survival to discharge [10]. Currently, the timing of terminating resuscitation is decided by each treating physician at their discretion.

Japan is an ideal setting to evaluate the actual association between CPR duration and outcome after OHCA. EMS personnel are not legally permitted to terminate resuscitation in prehospital settings in Japan. Therefore, resuscitation efforts must be continued until ROSC or hospital arrival is achieved. In Japan, several studies have assessed the impact of prehospital CPR duration for OHCA patients, but studies on in-hospital treatments such as care for post-cardiac arrest status as well as in-hospital CPR duration are limited [7–9].

The Japanese Association for Acute Medicine (JAAM)-OHCA Registry is a nationwide multicenter, prospective OHCA registry that collects both pre- and in-hospital data on resuscitation for OHCA patients and has enrolled approximately 23,800 non-traumatic adult OHCA patients between June 2014 and December 2017 [11]. Using this database, we aimed to assess the impact of CPR duration on 1-month survival with favorable neurological outcome.

## Methods

### Study design, patients, and settings

This study was a secondary analysis of a nationwide hospital-based prospective OHCA registry conducted by the JAAM. The detailed methodology of the JAAM-OHCA registry has been described previously [11]. In brief, prehospital data were collected by emergency medical service (EMS) personnel based on a Utstein-style template [12, 13], and in-hospital data collection was conducted by the physicians of each participating institution. The in-hospital data included information on the patients’ treatments, presumed etiology of arrests, and outcomes. This study included adult non-traumatic OHCA patients aged ≥ 18 years who were registered in the JAAM-OHCA registry between June 2014 and December 2017. We excluded those who did not have prehospital data, who were treated with extracorporeal membrane oxygenation, and those whose CPR duration was > 120 min or unknown. The ethics committee of each participating institution approved the study protocol.

### EMS system in Japan

A description of the EMS system in Japan has been provided elsewhere [14]. On receipt of an emergency call, an ambulance is dispatched from the nearest center. Emergency services are available for 24 h a day. Emergency life-saving technicians (ELSTs), who are the most highly trained emergency care providers, are permitted to insert an intravenous line and an adjunct airway for OHCA patients. Specially trained ELSTs are permitted to perform tracheal intubation and administer intravenous adrenaline. Basically, in each ambulance, there is a crew of three emergency providers with at least one ELST. CPR was conducted according to the Japanese CPR guidelines [15].

Importantly, termination of resuscitation (TOR) in prehospital settings by EMS personnel is not permitted because do-not-resuscitate orders (or living wills) are not acceptable in Japan [3, 15]. Therefore, almost all OHCA patients treated by EMS personnel are transported to hospitals, except in cases of decapitation, incineration, decomposition, rigor mortis, or dependent cyanosis [3, 15].

### Data collection and outcomes

The following data were obtained from the JAAM-OHCA registry: sex, age, cause of arrest, arrest witnessed by bystanders, bystander-initiated CPR, first documented rhythm, resuscitation time course, actual treatments in prehospital and hospital settings (e.g., epinephrine, TTM, PCI, and ECLS), and outcome data.

The causes of arrest were classified as cardiac or non-cardiac. The presumed cardiac cause category was determined by exclusion (i.e., the diagnosis was made when there was no evidence of a non-cardiac cause) based on Utstein-style guidelines. The diagnosis of cardiac or non-cardiac origin was clinically determined by the physician in charge.

The primary exposure was CPR duration. The definition of CPR duration was the time of professional CPR initiation to the time of ROSC or TOR, according to previous studies [5–9]. In the case that OHCA patients achieved ROSC before contact with EMS personnel, their CPR duration was regarded as 0 min, similar to previous studies [5–9]. Based on the results of previous studies, CPR duration was divided into the following categories: ≤ 20 min, 21–30 min, 31–40 min, 41–60 min, 61–90 min, and 91–120 min.

The primary outcome of this study was 1-month survival with favorable neurological outcome. Neurological outcomes were evaluated using the cerebral performance category (CPC) scale. Favorable neurological outcome was defined as a CPC 1 or 2 [12, 13]. The secondary outcome was 1-month survival.

### Statistical analysis

We compared patient characteristics, pre/in-hospital information stratifying outcome (1-month survival with CPC 1 or 2, 1-month survival with CPC 3–5, 1-month non-survival but achieved ROSC at least once, and non-ROSC) [6], using the Kruskal–Wallis rank test for continuous variables and Chi-square test for categorical variables.

We visually described the nonlinear relationship between CPR duration and the predictive probability of favorable neurological outcome using a restricted cubic spline with the univariable logistic regression model. In addition, to evaluate the association between CPR duration and each outcome after OHCA, univariable and multivariable logistic regression analyses were used to calculate crude or adjusted odds ratios (ORs) and their 95% confidence intervals (CIs). In the multivariable models, we adjusted for the following confounders: age (continuous), sex (male, female), cause of arrest (cardiac, non-cardiac), witnessed by bystanders (no, yes), bystander CPR (no, yes), first documented rhythm at the scene (shockable, non-shockable, presence of pulse), prehospital adrenaline administration (no, yes), prehospital advanced airway management (no, yes), and EMS response time (call to contact with patient). The covariates were preliminarily decided based on previous studies [6–9, 16].

In subgroup analyses, we aimed to evaluate the difference in the impact of CPR duration based on a variety of clinical features (witnessed arrests, first documented shockable rhythm, bystander CPR) or whether patients fulfilled the universal BLS TOR rule (not witnessed by EMS personnel, no prehospital shock, no prehospital ROSC) or ALS TOR rule (not witnessed by EMS personnel, no prehospital shock, no prehospital ROSC, not witnessed by bystanders, no bystander CPR). We illustrated the nonlinear relationship between CPR duration and the predictive probability of favorable neurological outcome using a restricted cubic spline with the univariable logistic regression model. In addition, we assessed the factors associated with favorable neurological outcome for patients who received prolonged CPR duration > 30 min (Ref) using multivariable logistic regression models with the covariates described above.

All statistical analyses were performed using SPSS statistical package (version 25.0 J, IBM Corp. Armonk, NY, USA) or R (R Foundation for Statistical Computing, version 3.4.3). All tests were two-tailed, and *P* values < 0.05 were considered statistically significant.

## Results

**Fig. 1:**
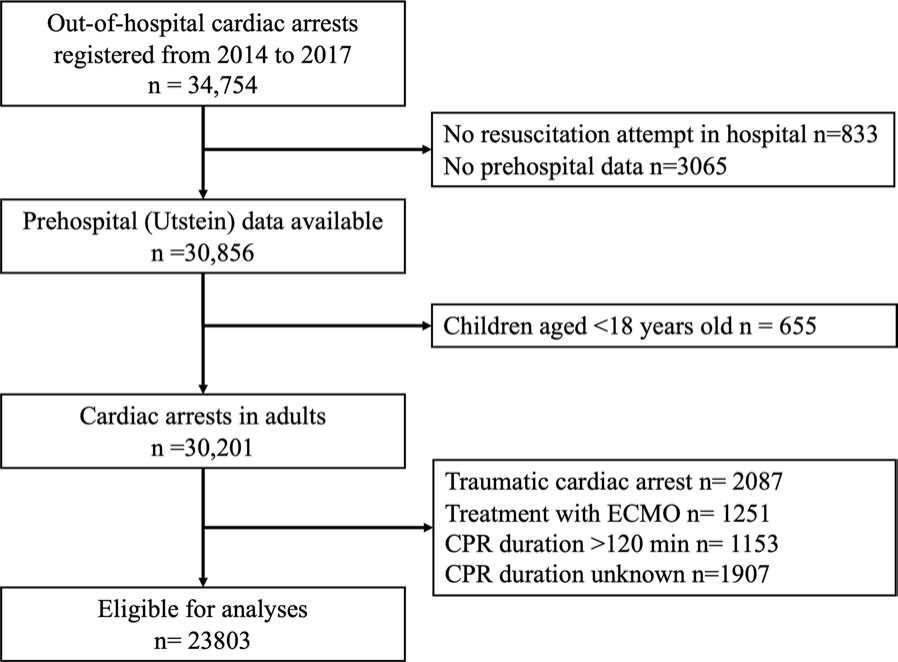
Patient flowchart. CPR: Cardiopulmonary resuscitation, ECMO: Extracorporeal membrane oxygenation

During the study period, 34,754 OHCA patients were registered in the JAAM-OHCA registry. After excluding those who met the exclusion criteria, 23,803 patients were eligible for our analyses (Fig. 1). There were 301 patients with 1-month survival with favorable neurological outcome (CPC 1 or 2), 386 patients with 1-month survival with unfavorable neurological outcome (CPC 3–5), 6337 non-survivors who achieved at least one ROSC, and 16,779 patients without ROSC (Table 1).

**Table 1:** Patient characteristics and pre-/in-hospital information among OHCA patients by outcomes

	Survival with CPC 1or 2 (*n* = 301)	Survival with CPC 3–5 (*n* = 386)	Non-survivor (*n* = 6337)	ROSC = 0 (*n* = 16,779)	*P* value
*Basic information*					
Men	226 (75.1)	243 (63.0)	3744 (59.1)	9710 (57.9)	< 0.001
Age, median (IQR) (Years)	62 (50–72)	71 (64–80)	78 (67–85)	78 (66–85)	< 0.001
Cardiac cause of arrest	272 (90.4)	187 (48.4)	2911 (45.9)	9899 (59.0)	< 0.001
*Prehospital information*					
Bystander witness	261 (86.7)	292 (75.6)	3875 (61.1)	5221 (31.1)	< 0.001
Bystander CPR	209 (69.4)	173 (44.8)	2827 (44.6)	7866 (46.9)	< 0.001
First documented rhythm at the scene					< 0.001
Ventricular fibrillation/pulseless ventricular tachycardia	85 (28.2)	83 (21.5)	362 (5.7)	678 (4.0)	
Pulseless electric activity/asystole	40 (13.3)	273 (70.7)	5611 (88.5)	15,707 (93.6)	
Presence of pulse	176 (58.5)	30 (7.8)	364 (5.7)	394 (2.3)	
Prehospital Epinephrine	26 (8.6)	110 (28.5)	1951 (30.8)	4271 (25.5)	< 0.001
Prehospital Airway management	45 (15.0)	199 (51.5)	3517 (55.5)	8980 (53.5)	< 0.001
EMS resuscitation times, median (IQR), (min)					
EMS response time (call to contact with a patient)	8 (6–10)	8 (6–9)	8 (7–10)	8 (7–10)	< 0.001
Hospital arrival time (call to hospital arrival)	30 (23–37)	28 (24–36)	32 (27–40)	33 (27–41)	< 0.001
*In-hospital information*					
First documented rhythm after hospital arrival					< 0.001
Ventricular fibrillation/pulseless ventricular tachycardia	70 (23.3)	30 (7.8)	171 (2.7)	400 (2.4)	
Pulseless electric activity/asystole	69 (22.9)	336 (87.0)	5771 (91.1)	16,379 (97.6)	
Presence of pulse	162 (53.8)	20 (5.2)	395 (6.2)	0 (0.0)	
Targeted temperature management	129 (42.9)	182 (47.2)	318 (5.0)	0 (0.0)	< 0.001
Coronary angiography	204 (67.8)	103 (26.7)	229 (3.6)	0 (0.0)	< 0.001
Percutaneous coronary intervention	99 (32.9)	51 (13.2)	100 (1.6)	0 (0.0)	< 0.001
*CPR duration, median (IQR), min*	0.0 (0.0–20.5)	31.3 (25.0–41.0)	35.0 (27.0–45.0)	40 (33–50)	< 0.001
90th percentile, min	32.8	41.0	57.2	74.0	
99th Percentile, min	58.9	81.1	103.0	108.0	
Maximum, min	67.0	94.0	120.0	120.0	

OHCA indicates out-of-hospital cardiac arrests; AED, automated external defibrillator; CPR, cardiopulmonary resuscitation; EMS, emergency medicine personnel; and IQR, interquartile range

Table 1 shows the patient characteristics and pre-/in-hospital information stratified by outcome. CPR duration, basic information, and pre-/in-hospital information differed according to the outcome. In patients with favorable neurological outcome, the 90th percentile, 99th percentile, and maximum CPR duration were 32.8, 58.9, and 67.0 min, respectively.

**Fig. 2:**
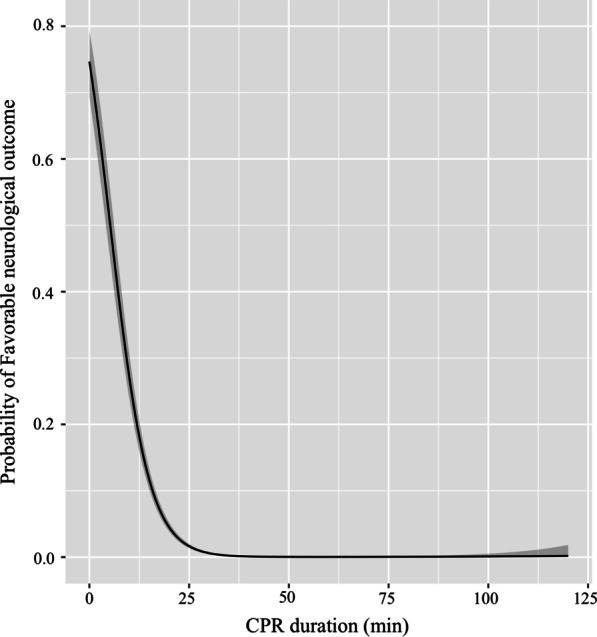
Probability of favorable neurological outcome by cardiopulmonary resuscitation duration

Table 2 shows the ORs of CPR duration for favorable neurological outcome. The proportion of patients with favorable neurological outcome was 20.8% (226/1084), 1.2% (37/3165), 0.4% (24/5466), 0.1%(12/9378), 0.1% (2/3990), and 0.0% (0/708) in the CPR duration ≤ 20 min, 21–30 min, 31–40 min, 41–60 min, 61–90 min, and 91–120 min groups, respectively. The probability of favorable neurological outcome decreased over time and dramatically after 20 min of CPR duration (i.e., ≤ 20 min vs 21–30 min, adjusted OR [AOR] 0.05; 95% CI, 0.03–0.06) (Table 2 and Fig. 2). A similar tendency was observed with regard to 1-month survival.

**Table 2:** Outcomes from OHCA according to CPR duration

	Total	Outcome (%)	Crude OR	(95% CI)	Adjusted OR	(95% CI)
*1-month survival*						
≤ 20 min	1084	349 (32.2)	Reference		Reference*	
21–30 min	3165	190 (6.0)	0.14	(0.11–0.16)	0.25	(0.20–0.31)
31–40 min	5466	93 (1.7)	0.04	(0.03–0.05)	0.08	(0.06–0.10)
41–60 min	9378	46 (0.5)	0.01	(0.01–0.01)	0.02	(0.013–0.026)
61–90 min	3990	8 (0.2)	0.004	(0.002–0.009)	0.005	(0.003–0.011)
91–120 min	708	1 (0.1)	0.003	(0.000–0.021)	0.003	(0.000–0.023)
*P* for trend^†^				< 0.001		< 0.001
*Favorable neurological outcome*						
≤ 20 min	1084	226 (20.8)	Reference		Reference*	
21–30 min	3165	37 (1.2)	0.05	(0.03–0.06)	0.15	(0.10–0.23)
31–40 min	5466	24 (0.4)	0.02	(0.011–0.03)	0.08	(0.05–0.12)
41–60 min	9378	12 (0.1)	0.005	(0.003–0.009)	0.02	(0.01–0.031)
61–90 min	3990	2 (0.1)	0.002	(0.000–0.008)	0.004	(0.001–0.016)
91–120 min	708	0 (0.0)	NA		NA	
*P* for trend^†^				< 0.001		< 0.001

OHCA indicates out-of-hospital cardiac arrest; OR, odds ratio; and CI, confidence interval

*Adjusted for age, sex, cardiac etiology of arrest, bystander witness, bystander CPR, first documented rhythm at the scene, prehospital advanced airway management, prehospital adrenaline administration, EMS response time

^†^CPR duration treated as continuous variable

**Fig. 3:**
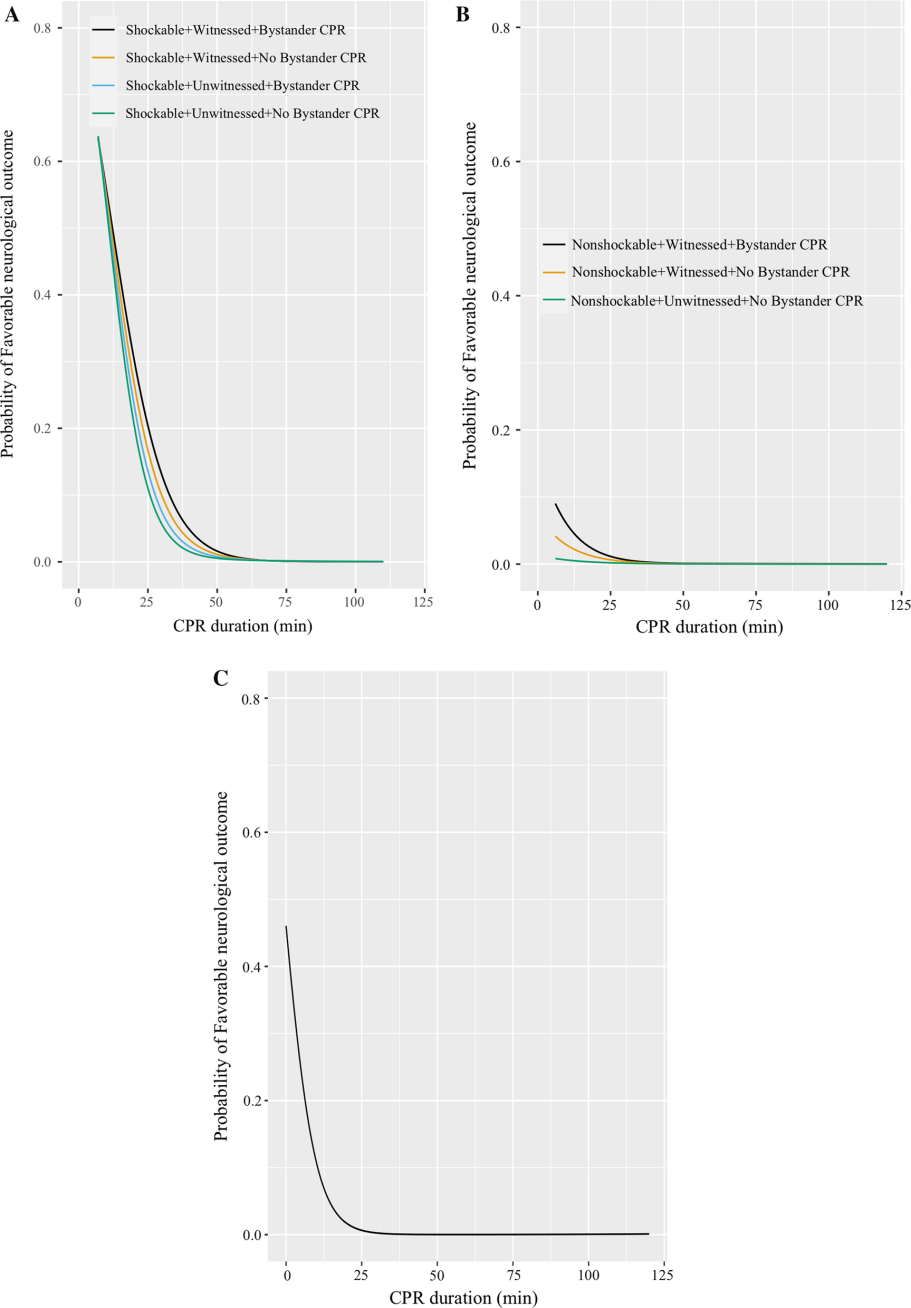
Probability of favorable neurological outcome by cardiopulmonary resuscitation duration for those **A** with shockable first documented rhythm, stratified witnessed status, and the presence of bystander CPR; **B** with non-shockable first documented rhythm, stratified witnessed status, and the presence of bystander CPR; and **C** those who fulfilled all items of BLS TOR rules. CPR: Cardiopulmonary resuscitation, BLS: Basic life support, TOR: Termination of resuscitation

Figure 3 presents the nonlinear relationship between CPR duration and favorable neurological outcome according to patient phenotype or those who fulfilled the universal BLS TOR. In almost all groups, longer CPR duration was associated with a decreased probability of favorable neurological outcome. However, the impact of CPR duration differed according to the observed groups (Fig. 3 and Additional file 1: Table). Importantly, only 2 of the 6632 patients who fulfilled the ALS TOR rule achieved favorable neurological outcome, and as a result, we could not describe the restricted cubic spline curve.

Table 3 shows factors associated with favorable neurological outcome after receiving prolonged CPR duration > 30 min. Younger age, bystander witness (vs unwitnessed), and the first documented shockable rhythm at the scene (vs non-shockable rhythm) were significantly associated with favorable neurological outcome (AOR for one increment age: 0.96, 95% CI: 0.94–0.98; AOR: 7.66, 95% CI: 3.04–19.30; and AOR: 14.70, 95% CI: 6.62–32.62, respectively).

**Table 3:** Factors associated with favorable neurological outcome after prolonged CPR duration

	Total	Cases (%)	Adjusted OR	(95% CI)
CPR duration (continuous variable)			0.92	(0.88–0.95)
Age (per one year increment)			0.96	(0.94–0.98)
Sex				
Men	11,494	24 (0.2)	0.73	(0.37–1.48)
Women	8060	14 (0.2)	Reference	
Cause				
Cardiac etiology	11,108	23 (0.2)	0.86	(0.41–1.80)
Non-cardiac etiology	8446	15 (0.2)	Reference	
Bystander witness				
No	11,985	6 (0.1)	Reference	
Yes	7569	32 (0.4)	7.66	(3.04–19.30)
Bystander CPR				
No	10,569	28 (0.3)	Reference	
Yes	8985	10 (0.1)	0.48	(0.23–1.02)
EMS response time (continuous variable)			1.04	(0.97–1.12)
First documented rhythm at the scene				
Non-shockable	18,104	18 (0.1)	Reference	
Shockable	944	16 (1.7)	14.70	(6.62–32.62)
Other	506	4 (0.8)	2.81	(0.89–8.84)
Adrenaline				
No	14,016	33 (0.2)	Reference	
Yes	5538	5 (0.1)	0.36	(0.14–0.94)
Advanced airway management				
No	1689	4 (0.2)	Reference	
Yes	17,865	34 (0.2)	0.78	(0.27–2.28)

CPR, cardiopulmonary resuscitation; OR, odds ratio; CI, confidence interval

## Discussion

Using the nationwide prospective OHCA registry in Japan, we demonstrated that a longer CPR duration was associated with a decrease in the probability of favorable neurological outcome. In particular, a rapid decrease in the probability of favorable outcome was observed within the first 20 min of CPR. Among patients with favorable neurological outcome, the 90th and 99th percentiles of CPR duration were 32.8 and 58.9 min. Furthermore, the impact of CPR duration differed among patients with key features. Patients of younger age, those with first documented shockable rhythm at the scene and with bystander witness, may be potential candidates to benefit from prolonged CPR duration.

The overall CPR duration was much longer in this study than in reports from Western countries [5, 6, 17]. Surprisingly, approximately 20% (4698/23,803) of all included patients received CPR for more than 60 min. This may be partly attributed to the difference in prehospital emergency care systems, such as the presence of prehospital TOR. Furthermore, our observed CPR duration was also longer than that observed in studies from Japan. Japanese studies tended to focus on the impact of prehospital CPR duration [7–9]. Importantly, prehospital CPR strategy (“Scoop and run” or “Stay and play”) varied according to the situation and regions [18], suggesting the importance of including both of pre- and in-hospital CPR duration. Although most of the longer CPR duration efforts may be futile, extended CPR could minimize the risk of failure to achieve favorable outcomes by stopping CPR duration efforts prematurely. Therefore, our results with a larger number of OHCA patients and longer CPR duration efforts could provide an important clue to determining an appropriate CPR duration.

The reduced probability of favorable neurological outcome over increasing CPR duration was consistent with the results of previous studies [5–9]. However, while one study from North America that assessed the impact of total CPR duration demonstrated that the probability of favorable neurological outcome steadily decreased (i.e., by approximately 25–30% for a 0 min duration to approximately 0% for a 45 min duration in shockable rhythm) [6], a rapid reduction in the probability was observed within approximately the first 20 min, and only a few OHCA patients achieved favorable neurological outcome thereafter. The observed difference may be due to the higher prevalence of ROSC before EMS resuscitation (CPR duration = 0). In Japan, nationwide efforts to increase bystander interventions for OHCA have successfully improved their actual proportion, leading to an increased success rate of ROSC before EMS arrival [19, 20]. Moreover, a rapid reduction in the probability should be noted. Given that the first 20 min of CPR are considered to be primarily performed in the prehospital setting, there should be space to improve prehospital CPR quality, such as by increasing the number of prehospital care providers, allowing administration of intraosseous lines by EMS personnel, and implementing the protocol of “stay and play [1, 2, 21, 22].” In patients with favorable neurological outcome, the 90th percentile, 99th percentile, and maximum CPR duration were 32.8, 58.9, and 67.0 min, respectively, which were longer compared to reports from North America with total CPR duration and Japan with prehospital CPR duration [5, 6]. We consider that longer CPR duration could lead to the extension of time to achieve favorable outcome, suggesting the need for reconsideration of the appropriate CPR duration.

We demonstrated that the impact of CPR duration differed according to each patient’s phenotype. Those with shockable rhythm, witnessed arrests, and bystander CPR appeared to be the best prognostic indicators (Fig. 3A), similar to the results of previous studies [6]. For example, based on our results, at least 30 min of CPR efforts should be performed for all cases, and at least 40 min for subjects with these favorable case features. Among those who received prolonged CPR duration, younger age, shockable rhythm, and bystander witness were prognostic factors for favorable neurological outcome. In contrast, we observed that the ALS TOR rule had an excellent negative predictive value. Similarly, those with non-shockable rhythm and unwitnessed arrests had an extremely low probability of favorable outcome regardless of the duration of resuscitation efforts (Fig. 3C and Additional file 1: Table). Taken together, the duration of resuscitation efforts should be judged by considering these differences in clinical features.

The important question is whether the extension of CPR duration without survival is futile. Recently, support for families who have lost a loved one has been attracting more attention as one of the “Sudden cardiac arrest survivorships [23].” In this study, the prevalence of achieving ROSC at least once was higher than that in a previous study from North America, while the proportion of all other outcomes was lower [6], which might be due to the longer CPR duration in this study. The best care for families of patients with cardiac arrests is still debated [23]. For families, being able to share the last moments, however, brief, with “a living patient” is considered to be helpful for psychological support and the acceptance of the loss of a loved one, although no study has evaluated such effect. Although considering the limited resources, routinely prolonging resuscitation efforts is not practical, further studies regarding care for families are warranted.

Finally, considering the observed rapid reduction in the probability of favorable outcome over CPR duration, our observations suggest the need for an alternative resuscitation strategy, unless OHCA patients are not immediately responsive to conventional standard ACLS. Extracorporeal CPR (ECPR) is a novel alternative treatment, which is expected to provide additional time to treat reversible conditions of arrest and serve as a bridge for left ventricular assist device implantation or cardiac transplantation [1, 2]. A recent randomized controlled trial demonstrated that early ECMO facilitated CPR for OHCA patients with refractory shockable rhythm and significantly improved the survival rate [24]. However, the cost- and resource-intensive resuscitation strategies should be applied to selected patients. Previous studies have demonstrated that several factors, such as older age, longer low flow duration, and initial non-shockable rhythm, were consistently found to be associated with worse prognosis [25–27]. Currently, ILCOR mentioned the need for research into which patient phenotypes benefit from ECPR [28]. Furthermore, taking the time dependence of the effectiveness of ECPR into consideration, when and how to effectively change the resuscitation strategy should be investigated to improve outcomes after OHCA.

## Limitation

Some inherent limitations of this study should be noted. First, we could not evaluate the quality of CPR and the resuscitation protocol of each EMS personnel and hospital. Second, a potential bias due to local EMS culture or guidelines (e.g. TOR not allowed) influences resuscitation efforts duration and outcome, and our results are therefore not generalizable to other regions. However, our findings from a unique prehospital care system could be used to accurately evaluate the impact of CPR duration and allow other countries to reconsider the length of resuscitation efforts. Finally, this hospital-based registry did not include all patients with OHCA in Japan. However, as this registry enrolled all consecutive OHCA patients transported to the participating institutions, the selection bias of this study is considered to be small.

## Conclusion

From a nationwide OHCA registry in Japan, we demonstrated that the probability of favorable neurological outcome rapidly decreased within a few minutes of CPR duration, but that the impact of CPR duration may be influenced by each patient’s clinical feature.

## Supplementary Information

**Additional file 1**. Outcomes from OHCA according to CPR duration and patient phenotype.

## Data Availability

Please contact the author for data requests.

## Abbreviations

OHCA: Out-of-hospital cardiac arrest
CPR: Cardiopulmonary resuscitation
ROSC: Return of spontaneous circulation
JAAM: The Japanese Association for Acute Medicine
EMS: Emergency medical service
FDMA: Fire and Disaster Management Agency
ELST: Emergency life-saving technician
AAM: Advanced airway management
AED: Automated external defibrillator
CPC: Cerebral performance category
TOR: Termination of resuscitation
IQR: Interquartile range
OR: Odds ratios
CI: Confidence interval
ECPR: Extracorporeal cardiopulmonary resuscitation
